# COVID-19 mortality in an area of northeast Brazil: epidemiological characteristics and prospective spatiotemporal modelling

**DOI:** 10.1017/S0950268820002915

**Published:** 2020-12-01

**Authors:** L. A. Andrade, D. S. Gomes, S. V. M. A. Lima, A. M. Duque, M. S. Melo, M. A. O. Góes, C. J. N. Ribeiro, M. V. S. Peixoto, C. D. F. Souza, A. D. Santos

**Affiliations:** 1 Graduate Nursing Programme, Federal University of Sergipe, Aracaju, Sergipe, Brazil; 2 Collective Health Research Center, Federal University of Sergipe, Aracaju, Sergipe, Brazil; 3 Graduate Programme in Parasitic Biology, Federal University of Sergipe, Aracaju, Sergipe, Brazil; 4 Department of Nursing, Federal University of Sergipe, Lagarto, Sergipe, Brazil; 5 Department of Occupational Therapy, Federal University of Sergipe, Lagarto, Sergipe, Brazil; 6 Sergipe State Department of Health, Aracaju, Sergipe, Brazil; 7 Department of Medicine, Federal University of Sergipe, Aracaju, Sergipe, Brazil; 8 Federal Institute of Education, Science and Technology of Sergipe, São Cristóvão, Sergipe, Brazil; 9 Department of Speech Therapy, Federal University of Sergipe, Aracaju, Sergipe, Brazil; 10 Department of Medicine, Center for the Study of Social and Preventative Medicine, Federal University of Alagoas, Arapiraca, Alagoas, Brazil

**Keywords:** COVID-19, mortality, pandemic, space–time clusters, spatial analysis

## Abstract

This study aimed to analyse the spatial–temporal distribution of COVID-19 mortality in Sergipe, Northeast, Brazil. It was an ecological study utilising spatiotemporal analysis techniques that included all deaths confirmed by COVID-19 in Sergipe, from 2 April to 14 June 2020. Mortality rates were calculated per 100 000 inhabitants and the temporal trends were analysed using a segmented log-linear model. For spatial analysis, the Kernel estimator was used and the crude mortality rates were smoothed by the empirical Bayesian method. The space–time prospective scan statistics applied the Poisson's probability distribution model. There were 391 COVID-19 registered deaths, with the majority among ⩾60 years old (62%) and males (53%). The most prevalent comorbidities were hypertension (40%), diabetes (31%) and cardiovascular disease (15%). An increasing mortality trend across the state was observed, with a higher increase in the countryside. An active spatiotemporal cluster of mortality comprising the metropolitan area and neighbouring cities was identified. The trend of COVID-19 mortality in Sergipe was increasing and the spatial distribution of deaths was heterogeneous with progression towards the countryside. Therefore, the use of spatial analysis techniques may contribute to surveillance and control of COVID-19 pandemic.

## Introduction

The coronavirus disease 2019 (COVID-19), caused by severe acute respiratory syndrome coronavirus 2 (SARS-CoV-2), first emerged in Wuhan city, China, and quickly became a threat to public health worldwide. The infection shows a high potential for spreading and has reached several countries, resulting in being declared as a pandemic by the World Health Organization (WHO) on 11 March 2020 [1].

The pandemic process has caused economic and social consequences in many regions around the world. Additionally, due to the quick dissemination of the virus, as well as the failures in the implementation of strategies to face it in some areas, the number of individuals infected and dead by the disease is increasing and this situation represents a challenge to health systems [1, 2].

According to the WHO, COVID-19 has affected more than 215 countries with approximately 50 million confirmed cases and 1 200 000 deaths worldwide by 10 November 2020, which corresponds to a mean case-fatality rate of approximately 2.5% [1]. In November 2020, the United States was the country most affected by COVID-19, with more than 10 million cases and 238 000 deaths. In Europe, one of the most affected continents, United Kingdom, Italy, France and Spain were the countries that registered the most deaths in the region [3].

By 23 July 2020, Brazil was the second country in the worldwide ranking of number of cases and deaths, and hence, considered the disease epicentre in Latin America [1], accounting for more than 2 million cases and 80 000 deaths. COVID-19 in the Northeast region of Brazil represents a serious public health problem and its impact may be greater, considering the interiorisation process and its growing expansion to more vulnerable areas [4]. In this region, there were high rates of incidence (1150) and mortality (41.4) per 100 000 inhabitants, which corresponds to the second most affected region in the country. The state of Sergipe has followed the exponential increase of rates, with a higher incidence and mortality than the regional (2288 cases and 57.2 deaths per 100 000 inhabitants, respectively) [5].

COVID-19 has a heterogeneous geographic dynamic in different regions. Although SARS-CoV-2 has a high potential for contamination and reaches all communities, some areas may be more affected than others, as many regions do not have adequate health resources and infrastructure to face the pandemic effectively [6].

In addition, the elderly and individuals with chronic diseases are at a higher risk for developing more severe forms and dying due to COVID-19 [7]. Consequently, specific characteristics of the population and the balance between supply and demand in the assistance of severe cases can lead to variations in local mortality rates [8, 9].

Since the first cases of COVID-19 in Brazil, spatial analysis techniques using geographic information systems (GIS) have been used to map the spread and severity of the pandemic [10, 11]. The use of this epidemiological tool enables the identification of the spatial dynamics of the disease, assisting in the planning, management and evaluation of public health policies [10].

Therefore, the aim of this study was to analyse the spatiotemporal distribution of deaths by COVID-19 in the state of Sergipe, Northeast, Brazil.

## Methods

### Study design

We carried out an ecological study using spatial analysis and temporal trend techniques. All COVID-19 deaths registered in Sergipe, as reported by the Sergipe State Department of Health (SES/SE), from 2 April to 14 June 2020 were included. The analysis units were the 75 municipalities of Sergipe and data were collected daily, considering the places of residence of the individuals deceased due to COVID-19.

### Study area description

Sergipe is located on the coast of the Northeast region of Brazil and has 75 municipalities divided into mesoregions (Leste – East, Agreste and Sertão – Hinterland). The metropolitan area includes Aracaju, its capital, Barra dos Coqueiros, Nossa Senhora do Socorro and São Cristóvão. Sergipe has a population of approximately 2 298 696 inhabitants, encompasses an area of 21 910 424 km^2^ and a demographic density of 94.3 inhabitants/km^2^ [12].

### Variables and measures

The variables analysed in the study were:

(a) COVID-19 deaths registered in the 75 municipalities of Sergipe;
(b) daily mortality rates in the state of Sergipe (per 100 000 inhabitants; considering the number of daily deaths by COVID-19 in the state as the numerator and the corresponding population as the denominator);
(c) weekly mortality rates (per 100 000 inhabitants; considering the number of weekly deaths by COVID-19 in Sergipe and the municipalities that compose the metropolitan and countryside regions as the numerator and their corresponding populations as the denominator) and
(d) accumulated mortality rate (per 100 000 inhabitants; considering the number of accumulated deaths by COVID-19 in the state and in each municipality as the numerator and the corresponding population as the denominator).

### Exploratory data analysis

The epidemiological variables used in the descriptive analysis were: age, sex, age group, race/colour, comorbidities, educational level, death site, time between symptom onset and test result, time between symptom onset and death and time between hospitalisation and death. Categorical variables were calculated by absolute and relative frequencies and continuous variables were calculated by median and interquartile range (IQR).

### Time trend analysis

The temporal trend analysis was performed using the segmented log-linear regression model. Weekly mortality rates of COVID-19 were considered dependent variables and epidemiological weeks were considered independent variables. The Monte Carlo permutation test was used to select the best model for inflection points, applying 999 permutations and considering the highest residue determination coefficient (*R*^2^) [13, 14].

The calculation to describe and quantify the trends was developed by percentage increments (APCs, annual percentage changes) and 95% confidence intervals (CIs). If more than one significant inflection was found during the study period, average annual percentage changes (AAPCs) were calculated. The trends were considered statistically significant when the APCs exhibited *P* < 0.05 and corresponding 95% CI did not include zero. Positive and significant values of APC indicate an increasing trend; negative and significant as a decreasing trend, and non-significant trends as stable, regardless of APC values [13, 14].

### Spatial cluster analysis

The addresses of the individuals deceased due to COVID-19 in Sergipe were georeferenced through the capture of latitude and longitude coordinates provided by Google Maps [15] and subsequent application of the non-parametric Kernel intensity test [16]. A density surface was generated for the visual detection of hot spots through statistical smoothing, indicating spatial cluster and continuous surface from georeferenced data [16].

Crude mortality rates were used in data analysis, as well as the local empirical Bayesian estimator. The Bayesian approach was used to minimise the instability caused by the random fluctuation of the cases. The model was applied to smooth the rates through weighted averages and create a new corrected coefficient [16]. The crude and smoothed rates were shown on thematic maps stratified into five categories of equal intervals: (a) 0 (without case records or information), (b) 0.1–10, (c) 10–20, (d) 20–30 and (e) ⩾30.

### Prospective spatiotemporal cluster analysis

The prospective space–time scanning statistic was performed to identify high-risk space–time clusters for COVID-19 mortality, using the discrete Poisson probability distribution model [17, 18]. The analysis identified potential active clusters during the study period. We consider still occurring space–time clusters that are present as active.

The null hypothesis (H0) is that the expected number of deaths by COVID-19 in Sergipe is proportional to the size of its population and reflects a constant risk. The alternative hypothesis (H1) is that the observed number of deaths exceeds the expected [17–19].

We established the following conditions for the model: minimum aggregation time of 2 days; minimum of five deaths; without overlapping clusters; circular clusters; maximum size of the spatial cluster of 10% of the at-risk population and maximum size of the temporal cluster of 50% of the study period. The primary and secondary clusters were detected using the log-likelihood ratio (LLR) test and represented through mapping. We calculated the relative risks (RRs) of mortality by COVID-19, taking into account each municipality and clusters in relation to its neighbours. The results that showed *P* < 0.05 using 999 Monte Carlo simulations were considered significant [17–19].

### Software

Microsoft Office Excel 2017 (Microsoft, Washington, USA) was used for data tabulation and descriptive analysis. Joinpoint Regression Program v. 4.5.0 (National Cancer Institute, Maryland, USA) [13, 14] was used to analyse the temporal trend. QGis 3.4.11 (QGIS Development Team, Open Source Geospatial Foundation Project, Oregon, USA) assisted in the creation of choropleth maps. TerraView 4.2.2 (Instituto Nacional de Pesquisas Espaciais, INPE, São José dos Campos, SP, Brazil) was used to carry out spatial analysis [20] and SaTScan™ 9.6 (Harvard Medical School, Boston and Information Management Service Inc., Maryland, USA) for the execution of space–time scanning [21].

### Ethical considerations

The study was approved by the Human Research and Ethics Committee of the Federal University of Sergipe (CEP/UFS) with acceptance number 4086909, following national ethical recommendations and the Declaration of Helsinki.

## Results

We observed the occurrence of 391 deaths due to COVID-19 in Sergipe, with an average of 130.3 deaths monthly. The median age was 65 years (IQR 53–77). The median time between the onset of symptoms and the result of diagnostic testing was 7 days (IQR 4–11) and between the onset of symptoms and death was 10 days (IQR 5–16). In cases that required hospital care, the median time between admission and death was 5 days (IQR 1–11). The highest percentage of deaths occurred among individuals aged ⩾60 years (62%), males (53%), non-white races/colours (58%) and those with low or no educational level (52%). The most frequent comorbidities were hypertension (40%), diabetes mellitus (DM) (31%) and cardiovascular disease (15%). The most prevalent location of death was a hospital (87%) (Table 1).

**Table 1.** Epidemiological characteristics of deaths by COVID-19, Sergipe, Brazil, 2020

Variables	Total (*n* = 391)
Age (years)	65^a^ (53–77)^b^
Time between symptom onset and death (days)	10^a^ (5–16)^b^
Time between symptom onset and test result (days)	7^a^ (4–11)^b^
Time between hospitalisation and death (days)	5^a^ (1–11)^b^
Age group, *n* (%)	
<60	148 (38)
⩾60	243 (62)
Sex, *n* (%)	
Male	208 (53)
Female	183 (47)
Comorbidities, *n* (%)	
Hypertension	157 (40)
DM	122 (31)
Cardiovascular diseases	60 (15)
Obesity	30 (8)
Kidney disease	28 (7)
Lung disease	22 (6)
Cancer	19 (5)
Brain stroke	15 (4)
HIV/AIDS	3 (1)
Race/colour, *n* (%)	
Brown	171 (44)
White	55 (14)
Black	36 (9)
Asian	19 (5)
Missing	110 (28)
Educational level, *n* (%)	
Illiterate	70 (18)
Elementary school	133 (34)
High school	57 (15)
Higher education	16 (4)
Missing	115 (29)
Marital status, *n* (%)	
Not married	103 (26)
Married	109 (28)
Widower	46 (12)
Divorced	25 (6)
Missing	108 (28)
Death site, *n* (%)	
Hospital	340 (87)
Other health units	40 (10)
Residence	8 (2)
Missing	3 (1)

*n*, absolute frequency; %, percentage.

a Median.

b IQR.

**Fig. 1.**
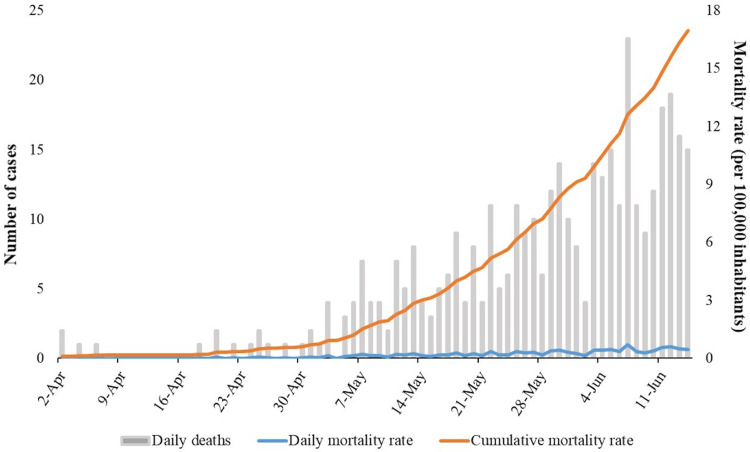
Number of deaths, daily mortality rates and accumulated mortality rates by COVID-19 in Sergipe, Brazil, from 2 April to 14 June 2020.

Figure 1 displays the number of deaths, and daily and accumulated mortality rates at the state level. The first death records occurred on 2 April 2020 and since then, there was an increase in the number of daily deaths, with the highest record on 7 June 2020 (*n* = 23). Therefore, there was an increase in the accumulated mortality rate, ranging from 0.09 (on 2 April 2020) to 16.91 (on 14 June 2020) per 100 000 inhabitants.

Table 2 displays the temporal trend of weekly mortality rates in Sergipe, its metropolitan area and countryside. The mortality rate in Sergipe showed an increasing trend (AAPC = 3.8, 95% CI 0.3–6.8, *P* < 0.01). In addition, increasing trends were observed when the analysis was performed by subgroup (metropolitan area and countryside), with a higher increase recognised to occur in countryside (APC = 3.8, 95% CI 3.0–4.5, *P* < 0.01) in comparison with the metropolitan area (AAPC = 2.9, 95% CI 0.2–5.5, *P* < 0.01).

**Table 2.** Temporal trends of the mortality rate of COVID-19 in the state of Sergipe, stratified by metropolitan and countryside

Region	Segmented period				Entire period		
	Days	APC (95% CI)	*P*-value	Trend	AAPC (95% CI)	*P*-value	Trend
Entire state	1–10	−14.9 (−32.4 to 7.1)	0.2	Stable	3.5* (0.3–6.8)	<0.001	Increasing
	10–36	10.3* (5.8–14.9)	<0.001	Increasing			
	36–74	3.8* (2.9–4.6)	<0.001	Increasing			
Metropolitan area	1–12	−12.1 (−24.6 to 2.5)	0.1	Stable	2.9* (0.2–5.5)	<0.001	Increasing
	12–47	7.9* (5.5–10.4)	<0.001	Increasing			
	47–74	3.1* (1.6–4.5)	<0.001	Increasing			
Countryside	1–74	3.8* (3.0–4.5)	<0.001	Increasing			

*Note*: Crude mortality rate (per 100 000 inhabitants).

APC, annual percentage change; AAPC, average annual percentage change.

*Significative trend *P*-value <0.001.

**Fig. 2.**
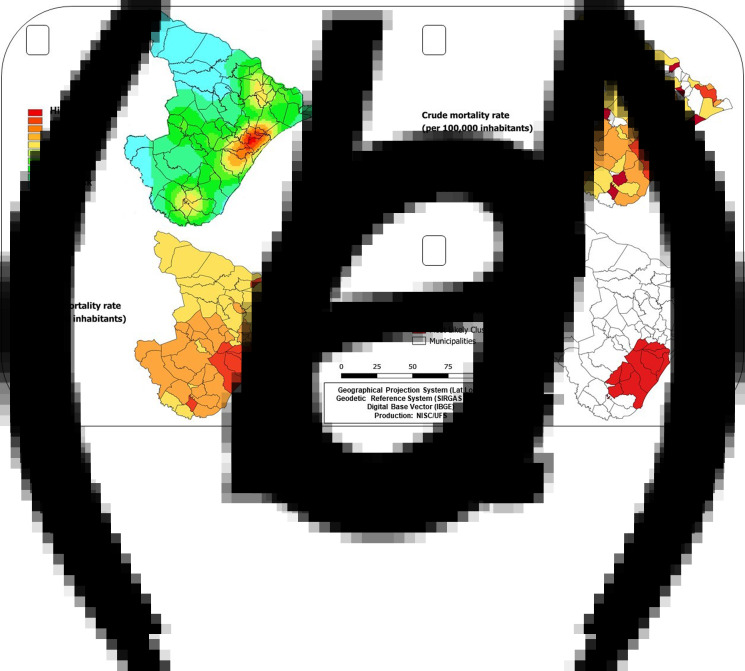
Spatial and spatiotemporal analysis of deaths by COVID-19 in the State of Sergipe, Brazil. (A) Kernel density map of deaths confirmed by COVID-19 in Sergipe, (B) distribution of crude mortality rates, (C) distribution of smoothed mortality rates by the estimated local empirical Bayesian and (D) analysis of space–time scanning in Sergipe.

The Kernel analysis identified a density high cluster (hot spot) of deaths located in the East mesoregion of Sergipe, represented by municipalities from the metropolitan area and neighbouring cities. Intermediate risk agglomerations were observed in the north and south regions of the state (Fig. 2A).

The distribution of crude and smoothed mortality rates by COVID-19 is shown in Figure 2B and C, respectively. When analysing crude rates, there was a dispersion of municipalities with high mortality located in the Northeast, West and South, besides a group formed by municipalities with high mortality rates located in the East mesoregion of the state. Considering the smoothed rates, a process of expansion of the mortality high cluster was found, compared to the crude rates. The cluster included the metropolitan area and neighbouring municipalities, as demonstrated in the Kernel analysis.

Figure 2D shows the emergent spatiotemporal cluster between 17 May and 14 June 2020, and comprises the municipalities of Itaporanga d'Ajuda, São Cristóvão, Salgado, Estância, Aracaju, Nossa Senhora do Socorro, Areia Branca, Boquim, Laranjeiras and Arauá. The RR of these municipalities was 5.66 (*P* < 0.001) (Table 3), indicating that more deaths were observed than expected.

**Table 3.** Space–time cluster of COVID-19 mortality from 2 April to 14 June 2020, Sergipe, Brazil

Duration (days)	Local	Expected	Observed	RR	LLR	*P*-value
May 17–June 14	Itaporanga d'Ajuda, São Cristóvão, Salgado, Estância, Aracaju, Nossa Senhora do Socorro, Areia Branca, Boquim, Laranjeiras, Arauá	75.76	225	5.66	138.61	<0.001

RR, relative risk for the cluster compared with the rest of the region; LLR, log-likelihood ratio.

## Discussion

Despite international, national and local efforts to face the pandemic, COVID-19 continues to cause socioeconomic damage and imply thousands of lives lost across the planet [1]. To the best of our knowledge, this is the first study to provide information about COVID-19 mortality in Sergipe applying temporal trend, spatial and spatiotemporal scan analysis techniques. Our findings can be useful for planning and developing strategies that aim to reduce deaths by COVID-19.

Our results suggest that Sergipe is challenging an important public health crisis. Thus, the state is configured as a potential risk area for mortality by SARS-CoV-2, since it exceeds the number of deaths at regional and national levels [5]. We observed an increase in the number of daily deaths and accumulated mortality rates from the first days of May 2020, which continued until the end of the analysed period.

The increase in the mortality rate persisted, even after the implementation of containment and mitigation strategies of the government initiated on 16 March 2020, such as school and university closures, suspension of events, concerts, sports activities and economic activity restrictions [22]. Failures in the mitigation process, mainly related to the inefficiency of social distancing strategies, could explain the maintenance of transmission and, therefore, the growth in the absolute number of deaths and mortality rates in the state [1, 2].

The association between increased cases and failures in disease mitigation strategies is reinforced by an investigation that identified an inverse relationship between social isolation rates and new cases of COVID-19 in Sergipe. A progressive decrease in social isolation was observed on 30 March 2020, and an increase in the daily average of accumulated cases noted in May 2020. In addition, the isolation rate on 13 May 2020 (42.2%) was lower than ideal (52.3%) to slow down the progression of the disease in the state [23].

Our findings indicate an increasing trend in weekly mortality rates due to COVID-19 across the state. The largest increase was observed in the countryside, compared to the metropolitan area. This is a serious concern for public administrators, as most municipalities in the countryside do not have sufficient infrastructure or intensive care unit (ICU) availability, which are essential. In addition, the high demand for reference hospitals in the metropolitan area can lead to overcrowding and consequent collapse of health services [24–26].

Deaths were more frequent among the elderly, according to corroborating studies conducted in China and Spain that also identified a greater prevalence of deaths in this age group and comparable median ages (65.8 and 67 years old, respectively) [27, 28]. Old age is constantly reported as a risk factor for fatal outcomes of COVID-19 [29, 30]. This suggests that senescence of the immune system may facilitate a faster progression of viral infection in these individuals [31]. Although the majority of deaths occurred in the elderly, we highlight that more than one-third of the deaths were among people under 60 years old. In this sense, risk reduction measures should not only be employed in vulnerable age groups [1].

We identified a higher proportion of deaths among males, consistent with findings of previous investigations that also showed this pattern of mortality [27, 32]. The predominance of severe outcomes of COVID-19 among men can be explained, generally due to this population's predisposition to adopt unhealthy lifestyles and have a higher prevalence of comorbidities [33]. Besides that, women are possibly less susceptible to viral infections due to the protective effects of the X chromosome and sex hormones, which have essential roles in the immune response [33, 34].

DM, hypertension and cardiovascular disease were the most frequent comorbidities. Previous studies [27, 32] identified a higher occurrence of fatal outcomes in people with these underlying diseases. The vulnerability of these patients may be related to changes in the immune response due to the pathophysiology of chronic diseases, resulting in increased susceptibility to complications [35].

A low educational level was reported in most of the deaths. This situation can make it difficult to understand health-related information in the context of a pandemic or to have basic good hygiene habits, implying that the impact caused by SARS-CoV-2 may be influenced by social determinants and vulnerable populations may be affected more. Although the pandemic process initiated among the upper social classes, COVID-19 has disproportionately affected the poorest [6]. A study conducted in New York identified higher rates of hospitalisation and death in a neighbourhood with a higher proportion of individuals with lower levels of education living in poverty [9].

The most deaths were registered to have occurred in hospitals, since the most severe cases required intensive hospital care for oxygenation and ventilation therapies, which are essential for treatment [36]. The median time between the onset of symptoms and death was 10 days. The infectious process and the development of severe disease appear, on average, 7 days after of symptom onset, and the time between of symptom onset and death varies from 2 to 8 weeks [37]. Regarding the time between hospital admission and death, our results are similar to those of another study, in which the median time was also found to be 5 days [32].

The COVID-19 pandemic is dynamic with heterogeneous geographic distribution. The first cases were registered in capital cities and metropolitan regions and quickly disseminated towards the countryside due to community transmission [11]. Moreover, economic networks, social mobility and travel flow are important factors to better understand the territorial progression of SARS-CoV-2 in different areas [38–40].

The spatial analysis techniques employed in this study allowed the identification of a mortality cluster by COVID-19 that comprises the metropolitan region and neighbouring cities. A local study [10] highlighted active clusters of the COVID-19 incidence in that same area of the state. The cluster of deaths in this region can be explained by the proximity of other municipalities to the metropolitan area. This region served as a source of spread of the virus to other neighbouring municipalities due to the intense urban mobility and economic activities between them. This may explain the higher incidence of the disease and, consequently, the high mortality in this area [10].

We identified, through prospective spatiotemporal analysis, an emerging cluster that includes municipalities of metropolitan area and cities that are parts of other regions of the state, such as the Agreste and Sul regions. Community transmission and virus dispersion in the countryside accrued increases in the number of deaths, since they lack the ideal structure for managing serious cases [11]. As a result, the high risk of mortality by COVID-19 in the countryside of Sergipe was expansive.

The study has limitations related to the use of secondary data, which may include problems inherent in underreporting deaths and the quality of information. However, the findings may provide subsidies for SARS-CoV-2, since the identification of high-risk areas allows better management of resources, such as structuring the flows of the health care network, training of care teams and availability of mechanical ventilators and intensive care beds. We also emphasise the need for further investigations to better understand the spatiotemporal dynamics, identify clinical and social determinants related to higher mortality due to COVID-19, and plan the safe reopening of economic activities in Sergipe.

## Conclusion

The study showed that COVID-19 mortality is a serious public health problem in Sergipe that has victimised the elderly, men and individuals with chronic diseases, such as hypertension, DM and cardiovascular disease. Sergipe showed an increasing trend of COVID-19 mortality throughout its territory, with accelerated growth in the countryside. Spatial analysis and space–time scanning revealed that the distribution of deaths is heterogeneous, with the presence of an active high-risk cluster for mortality in the metropolitan area and neighbouring cities. We highlight the need for greater targeting of disease prevention and control strategies in the main risk areas, aiming to reduce the growth curve of mortality from the disease in the state.

## Data Availability

The data that support the findings of this study will be available on request and permission of via e-mail from the corresponding author.
